# Deranged Biochemical Markers As Early Predictors for the Development of Hepatorenal Syndrome in Patients With Alcoholic Liver Cirrhosis

**DOI:** 10.7759/cureus.47927

**Published:** 2023-10-29

**Authors:** Durga Deorukhkar, Archana Sonawale, Aman Goyal, Kshitij Sonawale

**Affiliations:** 1 Department of Internal Medicine, Seth Gordhandas Sunderdas (GS) Medical College and King Edward Memorial (KEM) Hospital, Mumbai, IND

**Keywords:** biomarkers, nephrology, gastroenterology, prognosis, alcoholic liver cirrhosis, hepatorenal syndrome

## Abstract

Objective

To investigate predictive biomarkers correlated with the onset of hepatorenal syndrome (HRS) in individuals with alcoholic liver cirrhosis using various factors, including age, sex, and laboratory indicators such as serum sodium, bilirubin, PT/INR, and albumin levels. Additionally, we sought to establish a correlation between the occurrence of hepatic encephalopathy (HE), spontaneous bacterial peritonitis (SBP), and the model for end-stage liver disease (MELD) score at the time of diagnosis and the development of HRS in cirrhotic patients.

Methods

This cross-sectional study spanned 12 months and included a total of 83 patients as its sample size. This study was conducted at the Department of Internal Medicine, a tertiary care hospital situated in Mumbai, India. Two distinct groups were formed: one consisted of patients diagnosed with HRS, and the other group comprised patients with alcoholic liver cirrhosis but without HRS. This study aimed to investigate potential relationships with the suggested risk factors. To discern statistically meaningful distinctions among categorical variables, the chi-square test was employed, whereas for continuous variables, analysis of variance (ANOVA) was used. Only patients who provided written informed consent were included in this study.

Results

No correlation was found between patients with and without HRS with respect to age (p=0.056) and sex (p=0.067). The presence of HE (p<0.001), SBP (p=0.021), hyponatremia (p=0.0001), hypoalbuminemia (p<0.0001), higher PT/INR (p=0.03), and higher MELD score (p=0.0002) were found to be correlated with an increased risk of developing HRS. Hyperbilirubinemia was not correlated with an increased risk of developing HRS (p=0.157).

Conclusions

HRS is a severe and potentially avoidable complication associated with advanced liver cirrhosis, characterized by a notably high mortality rate. By closely monitoring key biomarkers, such as serum sodium, PT/INR, and albumin levels, in addition to assessing the presence of SBP and HE during the initial presentation of patients with alcoholic cirrhosis, medical professionals may be able to identify those at a heightened risk of developing HRS. This, in turn, enables the swift diagnosis and implementation of aggressive treatment strategies. Such measures not only hold the potential to reverse HRS but also enhance survival rates among individuals with alcoholic liver cirrhosis, thereby increasing the pool of candidates eligible for liver transplantation, which remains the cornerstone of treatment.

## Introduction

Hepatorenal syndrome (HRS) is defined as the onset of kidney failure in individuals with advanced liver disease, with no specific kidney-related abnormalities observed on histological examinations [1]. HRS is a known complication of patients with liver cirrhosis and ascites. From the first presentation of ascites in a patient, the five-year probability of developing HRS is 11%, with an increasing probability in patients who develop hyponatremia or refractory ascites [2]. Notably, studies have shown that kidney function typically improves and returns to normal after liver transplantation in such cases. Renal dysfunction is believed to be the most severe manifestation of the vascular and neurohumoral alterations associated with severe liver disease. Without liver transplantation and prior to recent studies of treatment using vasoconstrictors, recovery of renal function was rare, and prognosis was poor with a median survival of only two weeks [3].

Given the unrelenting poor outcome of HRS in cirrhotic patients, it is important to recognize and prevent the development of HRS if possible [4]. Our study aimed to assess independent risk factors correlated with the development of HRS in patients with alcoholic liver cirrhosis and evaluate their predictive value. These correlations are not well defined in the current literature, which was our aim. Our study aimed to aid physicians in monitoring patients at an increased risk of developing HRS, as prevention is often more effective than cure.

## Materials and methods

Study design 

This cross-sectional study was conducted over a period of 12 months, from June 2021 to June 2022, on a sample size of 83 patients. This study was conducted in the Department of Internal Medicine, in a tertiary care hospital in Mumbai, India.  

Inclusion criteria 

This study included patients aged 12 years and older who had alcohol-induced cirrhosis diagnosed through a combination of medical history, physical examination, and either ultrasonography or computed tomography. Only patients who provided written informed consent were included in this study. 

Exclusion criteria 

We excluded patients diagnosed with hepatitis B, hepatitis C, autoimmune hepatitis, Wilson's disease, metabolic liver diseases, as well as those with acute kidney injury resulting from acute febrile illness, acute gastroenteritis, renal hypoperfusion due to blood loss, and pre-existing chronic kidney disease. Additionally, we excluded patients with coronary artery disease, acute coronary syndrome, dilated cardiomyopathy, or chronic obstructive pulmonary disease with cor pulmonale. Pregnant and lactating women were excluded from this study.

Study procedure 

The details of the patients were recorded, including their medical history, as well as the findings from their physical and clinical examinations upon admission. Patients with alcoholic liver cirrhosis were selected and categorized into two groups: those diagnosed with HRS according to the International Club of Ascites's definition and those without an HRS diagnosis. Participants in both groups were assessed for age, sex, and the presence of hepatic encephalopathy (HE) and spontaneous bacterial peritonitis (SBP). Additionally, serum sodium levels, serum bilirubin levels, PT/INR levels, and serum albumin levels were also noted to identify prognostic laboratory markers for the development of HRS. In our study, hyponatremia was defined as serum sodium levels less than 135 mEq/L, hypoalbuminemia as a serum albumin level of less than 2.45 g/dL, and hyperbilirubinemia as serum total bilirubin levels greater than 1 mg/dL. The MELD score was calculated using the patient's INR, serum creatinine, and serum bilirubin levels [5,6]. The MELD score has been validated as a predictor of survival in patients with cirrhosis, alcoholic hepatitis, acute liver failure, and acute hepatitis, and we wanted to check its correlation with the development of HRS in patients [6].

Statistical analysis 

Statistical significance among continuous variables was determined using the t-test and analysis of variance (ANOVA). Differences in categorical variables were assessed using the chi-square test. The log-rank test was used to identify the predictors with the most significant independent influence on prognosis. Statistical significance was set at a p-value of less than 0.05, with a 95% confidence interval. Statistical analyses were conducted using the IBM SPSS software, version 28 (IBM Corp., Armonk, USA).

Ethical considerations 

Ethical approval was granted by the Institutional Ethics Committees (IEC-II) of Seth Gordhandas Sunderdas Medical College and King Edward Memorial Hospital. The approval number was EC/159/2019. The study adhered to the principles of the Declaration of Helsinki. All participants were selected after obtaining written Informed Consent.

## Results

A total of 83 patients with alcoholic liver cirrhosis were included in the study. Of the 83 patients, 41 (49.4%) fulfilled the criteria for HRS at the time of selection, and 42 (50.6%) were selected as controls without HRS.

Demographic distribution

The incidence of HRS in different age groups is shown in Table 1. No correlation was found between patients with and without HRS with respect to age (p-value: 0.056). It was observed that out of the 41 patients with HRS, 37 were male (87.95%) and 4 were female. Of the 42 patients who did not have HRS, 36 were male (85.71%) and the rest were females. No correlation was found between patients with and without HRS with respect to sex (p-value: 0.067).

**Table 1:** Distribution of patients with and without HRS by age group HRS, hepatorenal syndrome

Age group (in years)	Patients without HRS	Patients with HRS
≤30	4 (50%)	4 (50%)
31-40	17 (54.8%)	14 (45.2%)
41-50	14 (56%)	11 (44%)
51-60	4 (33.3%)	8 (66.7%)
61-70	2 (40%)	3 (60%)
>70	1 (50%)	1 (50%)

Prognostic markers for the development of HRS

It was found that all patients with HRS at the time of selection experienced HE (n=41), whereas out of the 42 patients who did not develop HRS, 40.5% (n=17) had HE, and 59.5% (n=25) did not. The presence of HE at the initial presentation was correlated with a higher risk of predicting HRS (p<0.001). Furthermore, it was found that out of the 41 patients with HRS, 63.4% had SBP and 36.6% did not have SBP at the time of participant selection. Among the 42 patients without HRS, only 38.1% (n=16) had SBP, and 61.9% (n=26) did not (Table 2). The presence of SBP at presentation correlated with a higher risk of predicting the development of HRS (p=0.021).

**Table 2:** Correlation between hepatic encephalopathy and spontaneous bacterial peritonitis at presentation and the development of HRS The number in parentheses denotes a percentage unless specified otherwise. HRS, hepatorenal syndrome

Variable	Patients with HRS (n=41)	Patients without HRS (n=42)	P-value
Hepatic encephalopathy (at presentation)	41 (100)	17 (40.5)	<0.001
Spontaneous bacterial peritonitis (at presentation)	26 (63.4)	16 (38.1)	0.021

In terms of laboratory markers, it was found that out of the 41 patients with HRS, 97.6% (n=40) had hyponatremia, while 2.4% (n=1) had normal serum sodium levels, with a mean serum sodium value of 120.83±6.33 mEq/L. Among the 42 patients without HRS, 42.9% (n=18) had hyponatremia, and the remaining 57.1% (n=24) had normal serum sodium levels, with a mean serum sodium of 131.71±4.27 mEq/L in the hyponatremia group (Table 3). The presence of hyponatremia was significantly correlated with a higher risk of predicting the development of HRS (p=0.0001). We also observed that of the 41 patients with HRS, 95.1% (n=39) had hypoalbuminemia (mean value=2.20 g/dL), whereas 4.9% (n=2) had normal serum albumin levels. Among the 42 patients without HRS, 69% (n=29) had hypoalbuminemia (mean value=2.67 g/dL), and the remaining had normal serum albumin levels. Hypoalbuminemia was significantly correlated with a higher risk of predicting the development of HRS (p<0.0001) (Table 3). Our study also observed that all 41 patients with HRS had hyperbilirubinemia (mean value=13.14±7.63 mg/dL), whereas out of the patients without HRS, 95.2% (n=40) had hyperbilirubinemia (mean value=3.53±2.54 mg/dL), and the remaining 4.8% (n=2) had normal bilirubin levels. Increased serum bilirubin levels were not significantly correlated with predicting the development of HRS (p=0.157) (Table 3).

**Table 3:** Correlation between the development of HRS and baseline laboratory marker levels The number in parentheses denotes a percentage unless specified otherwise. HRS, hepatorenal syndrome

Variable	Patients with HRS (n=41)	Patients without HRS (n=42)	P-value
Hyponatremia	40 (97.6)	18 (42.9)	0.0001
Hypoalbuminemia	39 (95.1)	29 (69)	<0.0001
Hyperbilirubinemia	41 (100)	40 (95.2)	0.157
High PT/INR ratio	35 (85.4)	19 (45.2)	0.03

When correlating with the PT/INR ratio, it was found that of the 41 patients with HRS, 85.4% (n=35) had a higher PT/INR ratio (mean value of 1.98±0.57), while only 14.6% (n=6) had a normal PT/INR ratio. Among the patients who did not develop HRS, 45.2% (n=19) had a higher-than-normal PT/INR ratio (mean value of 1.56±0.41), and the rest (n=23) had a normal ratio (Table 3). A higher PT/INR ratio was significantly correlated with a higher risk of predicting the development of HRS (p=0.03). Finally, we found that out of the 41 patients with HRS, four had an MELD score (Table 4) between 20 and 29, 29 had an MELD score between 30 and 39, and eight had an MELD score >40 (mean MELD score=35.10±4.73). Among the 42 patients without HRS, 22 had an MELD score between 10 and 19, 18 had an MELD score between 20 and 29, one had an MELD score between 30 and 39, and one had an MELD score >40 (mean MELD score=20.6±6.11). Thus, a higher MELD score at presentation was significantly correlated with a higher risk of predicting HRS's development (p=0.0002).

**Table 4:** Correlation between the MELD score and HRS HRS, hepatorenal syndrome

MELD score	<9	10-19	20-29	30-39	>40	Total number
Patients without HRS	0	22	18	1	1	42
Patients with HRS	0	0	4	29	8	41

## Discussion

HRS is a potentially preventable but serious complication of advanced liver cirrhosis and is associated with a high mortality rate [7,8]. Although several complex tools exist for risk assessment, prognostication, and prediction of mortality in liver cirrhosis, none exists for predicting HRS, which is universally considered the standard. Fortunately, several treatment strategies are currently available for decreasing the probability of HRS in patients with alcoholic liver cirrhosis. Recent advances in the medical management of this condition, particularly the use of vasoconstrictors with plasma volume expansion, have improved the gloomy outlook associated with this complication. Intravenous albumin infusion decreased the risk of renal impairment in patients with liver cirrhosis and SBP [9]. It is known that early identification and prompt initiation of treatment for HRS could lead to improved survival, hence making the pursuit of reliable prognostic markers an important field of study [7].

In a study conducted by Janicko et al., younger age was significantly correlated with an increased risk of developing HRS, but no correlation was found between the risk of developing HRS and sex [10]. In our study, no correlation was found between patients with or without HRS with respect to age or sex. We found a correlation between the presence of SBP at presentation and a higher risk of predicting HRS. In a clinical trial, primary prophylaxis against the development of SBP in patients with underlying liver cirrhosis was associated with a lower risk of developing HRS and higher survivability [11]. Thus, it may be prudent for physicians to adequately manage SBP if it is present, or even consider the use of antibiotics as prophylaxis in cirrhotic patients. Furthermore, our study found that the presence of HE at baseline was also a significant predictor for HRS. To the best of our knowledge, no other study has tested this correlation. However, in a study conducted by Greinert et al., no association was demonstrated in patients with HRS, whether they had HE or not, in the risk of complications [12]. This raises the question of the clinical relevance of patients with HE being more likely to develop HRS.

In our study, we found that hyponatremia correlated with predicting HRS. This finding is consistent with the results of a study conducted by Gines et al. [13]. Our study found a correlation between an increased risk of developing HRS and hypoalbuminemia and higher PT/INR levels. This finding is consistent with the results of a study conducted by Janicko et al. [10]. They also found that higher serum bilirubin levels were correlated with a higher risk of predicting the development of HRS, but our study found no such correlation.

Limitations

It is important to note a few limitations to our study. First, we observed that the majority of patients were male (87.95%). This could be attributed to the fact that only alcohol as an etiology of cirrhosis was considered in the inclusion criteria. Second, the small sample size and recruitment of patients from a single center limits the generalizability of our study. Finally, the diagnosis of HRS is primarily a diagnosis of exclusion, which may lead to an underdiagnosis of the condition. This may result in the potential introduction of bias in the results of the study and limit the real-world clinical application of our results. However, this limitation is shared by most studies on HRS.

Therapeutic modalities for hepatorenal syndrome

Abstinence from alcohol plays a critical role in the reversal and improvement of HRS in patients with alcoholic liver cirrhosis as the underlying etiology [14]. Other treatment options for HRS include the use of vasoconstrictors and albumin, which improve short-term survival and renal function, especially in patients awaiting liver transplantation [7,9]. Norepinephrine and terlipressin are equally effective vasoconstrictors, although terlipressin is associated with a higher risk of side effects and is more expensive [13]. Patients with HRS who fail to respond to medical therapy may require renal replacement therapy or transjugular intrahepatic portosystemic shunts as alternative therapies [15,16]. Simultaneous liver-kidney transplantation (SLK) is required in many patients to improve post-transplant outcomes [15,16]. However, the criteria for selecting patients who would benefit from SLK transplantation are based on consensus and lack compelling evidence to support them.

## Conclusions

Unfortunately, the predictors of HRS are much less studied, especially in India, owing to the shortage of adequate medical infrastructure and extensive research laboratories in non-metropolitan cities, under-reporting of symptoms by patients, and seeking treatment late in the disease course. Our study aimed to resolve this dilemma.

By closely monitoring biomarkers such as serum sodium, PT/INR, albumin levels, and the presence of SBP and HE at presentation in patients with alcoholic cirrhosis, physicians might be able to predict patients who are more likely to be at risk of developing HRS and subsequently make prompt diagnoses and institute aggressive treatment. Interestingly, we found that sex and age did not play a role in predicting the risk of HRS. This may help reverse the syndrome of HRS and improve survival rates in patients with alcoholic liver cirrhosis due to this complication, thus increasing the number of eligible liver transplant candidates, the mainstay of treatment.
